# No Correlation Between Ethical Judgment in Trolley Dilemmas and Vaccine Scenarios for Nurse Specialist Students

**DOI:** 10.1177/1556264620911234

**Published:** 2020-03-19

**Authors:** Gry Oftedal, Ingrid H. Ravn, Fredrik A. Dahl

**Affiliations:** 1 Department of Philosophy, Classics, History of Art and Ideas, University of Oslo, Norway; 2 Department of Nursing and Health Promotion, Oslo Metropolitan University, Norway; 3 Health Services Research Unit, Akershus University Hospital, Lørenskog, Norway; 4 Institute of Clinical Medicine, Campus Ahus, University of Oslo, Norway

**Keywords:** trolley problems, thought experiments, deontology, consequentialism, vaccine trials, research ethics, nurses

## Abstract

We tested whether responses to trolley problems by nurse specialist students correlated with their responses to hypothetical vaccine problems, as a follow-up to a similar study on ethics committees. No statistically significant correlation was found between the trolley and vaccination scores. These results confirmed and strengthened the finding of a very weak correlation (possibly zero), and the point estimate was even lower than for the ethics committees. Hence, the nurse specialists’ responses to the trolley problems cannot be used to indicate any direction for their responses to the vaccine problems, although there is a common core issue of sacrificing some for many. The respondents reported a relatively high willingness to push one man in front of a trolley to save five. They also reported a high willingness to act in trolley dilemmas compared with vaccination dilemmas, although the dimensions of risk–reward ratios and consent heavily favored the latter.

## Introduction

Dahl and Oftedal (2019) found in a study on 75 ethics committee members and staff that the respondents’ replies to classic trolley problems did not correlate with how they responded with regard to the ethical justifiability of a set of hypothetical vaccination trial scenarios. This article presents a follow-up study on a group of advanced nurse specialist students.

Trolley problems are classic thought experiments that are widely used in ethics and psychology and often seen as illuminating differences between consequentialist and deontological thinking (Foot, 1967; Kamm, 2007, 2015; Thomson, 1976, 1985, 1990; Unger, 1996). In most trolley problems, one has to choose between letting one or five people die under varying circumstances, which may trigger different intuitions, value systems, and/or psychological responses. In the original version, a trolley is on its way toward five people on a track. The question is whether you should save these five people by switching the trolley on to a second track, where a single person will be killed. A different version considers a similar scenario, but in this case, you are asked whether you should push a fat man from a bridge above the track to stop the train and save the five people on the track. In surveys, most people reply that the trolley should be switched in the original version, but much fewer hold that the fat man should be pushed (Banerjee et al., 2011; Tinghög et al., 2016). Thus, according to most people, the consequentialist conclusion, that it is morally better that one dies rather than five, is apparently trumped by a moral rule saying it is wrong to actively kill someone, even if it saves many from dying.

When testing new vaccines in research trials, there is a certain risk that research participants may be harmed by side effects. Research participants often take such risks and thereby contribute to the development of new vaccines that may save thousands or even millions of lives. Although vaccine trials in many respects are very different from trolley dilemmas, there is a common core issue of putting the well-being or lives of a few at risk to save a greater number of people. In literature central to the field, this parallel between trolley problems and vaccine scenarios is frequently recognized and discussed (Bartels, 2008; Bialek & De Neys, 2016; Rosenbaum, 2018; Wiss et al., 2015; Young & Koenigs, 2007) and is sometimes used as a basis for transferring ethical reasoning from trolley dilemmas to vaccine scenarios (Andrade, 2019; Rosenbaum, 2018; Spier, 2011). Similarly, such parallels are also used as a basis for transferring ethical reasoning from trolley dilemmas to other medical research ethics contexts, for instance, to the ethics of sham surgery and “human challenge studies” (Albin, 2005; Andrade, 2019; Fitzpatrick, 2003; Fritz, 2015; Hope & McMillan, 2004).

As argued by Plunkett and Greene (2019), trolley dilemmas “are best understood as high-contrast cognitive probes . . . that can dissociate processes within people, not as moral personality tests or surrogates for real-world emergencies” (p. 2). For our specific study, although there is an interesting common core issue in trolley dilemmas and vaccine scenarios, there are also several important differences between them that may affect moral judgment. Overall, vaccine dilemmas are realistic moral problems, which involve uncertainty and are embedded in a rich context, whereas trolley dilemmas are stripped of any context, and we know the outcomes with full certainty. There are also several other relevant differences between the two dilemma types discussed in Dahl and Oftedal (2019). However, as mentioned above, vaccine dilemmas are often interpreted to mirror important properties of trolley dilemmas, and trolley dilemmas are used in the literature to guide reasoning about vaccine trials and other medical ethics problems. A possible background for this use of trolley problems may be the possibility that people’s utilitarian–deontological orientation mapped in trolley problems may shine through in how they think about more realistic and context-rich moral problems, despite the mentioned differences. Some authors have found that people’s responses to trolley dilemmas do correspond to how they reply to more realistic dilemmas and dilemmas that involve different types of harm (Bostyn et al., 2019; Dickinson & Masclet, 2018; Gold et al., 2013). On this background, we find it relevant to provide empirical research on the possible link between how people judge trolley problems and how they judge more complex moral scenarios, particularly since such a link is assumed in relevant literature. Thus, the introduced differences between trolley problems and vaccine scenarios in the current study is a design choice with an aim to test whether intuitions mapped in trolley surveys may correlate with people’s responses to more realistic dilemmas with more contextual details. It is, however, important to keep in mind that trolley dilemmas were not originally meant for this type of use.

In a previous study on ethics committee members and staff, a link between responses to trolley dilemmas and vaccine scenarios was tested using a questionnaire, which included three classical versions of the trolley problem in addition to three different hypothetical vaccine trial scenarios (Dahl & Oftedal, 2019). The respondents were asked whether they should act in the trolley problems (switch the tracks or push the man) and whether they found the vaccine trial scenarios ethically justifiable. The vaccine trials considered different versions of the testing of an effective but potentially harmful vaccine to verify its safety for subsequent use in the general population. The hypothesis was that the participants’ replies to the trolley dilemmas would correlate with how they responded to the vaccine trial scenarios. However, the results showed no statistical correlation between the problem sets, so the participants’ responses to the trolley problems provided no information on their judgment tendencies in the hypothetical vaccine trials, and vice versa. In addition, there was a general high willingness to sacrifice one person to save five in the trolley dilemmas and a low willingness to consider the vaccine trials ethically justifiable. Compared with previous studies, there was also a high willingness to push the fat man in front of the train to save five people.

The previous study concluded that “the context sensitivity of moral judgement seems to be so high that barely any ‘core intuitions’ carry over from the simplified trolley case to the more contextual vaccine case” (Dahl & Oftedal, 2019, p. 30). One possible criticism is that the study was performed only on ethics committee members and staff. This group is particularly relevant, as they frequently evaluate and make decisions about the ethical justifiability of, for example, vaccine trials and are trained to conduct such evaluations. One may suspect, however, that this training makes them particularly careful and conservative with regard to allowing the hypothetical vaccine trials and may eschew the results in particular directions. It is therefore of interest to include different groups of respondents to investigate whether or not the results can be reproduced, which was the main goal for the present follow-up study on specialist nurses.

Advanced nurse specialists work in acute care settings and are a relevant group for this study, due to their frequent exposure to ethically sensitive situations. Ethical dilemmas in nursing practice include conflicts between consequentialist and deontological thinking and are often caused by end-of-life situations (Rainer et al., 2018). According to Oberle and Hughes (2001), decisions in end-of-life scenarios present significant ethical problems for both nurses and doctors, and differences between these two groups in how they describe such problems seem to be “a function of the professional role rather than differences in ethical reasoning or moral motivation” (Oberle & Hughes, 2001, p. 707).

In the following, we present methods and results of the follow-up study and discuss the lack of correspondence between replies to the two problems sets reproduced in the group of specialist nurse students. We address how the specialist nurse study confirms and strengthens the findings in the study on ethics committee members and staff.

## Method

The study was conducted in 2017 during a course in Theory of Science and Research Methods for nurses in advanced programs of Anesthesia-, Pediatric-, Intensive Care-, and Theater Nursing (APIT) at Oslo Metropolitan University. One of the admission criteria for the program was that the nurse had worked at least 2 years in the specialist health service.

The specialist nurses replied to a questionnaire with six yes/no questions. As in Dahl and Oftedal (2019), the three first items were grouped into a Trolley index, and the three last into a Vaccination index, and we defined the index scores to be the number of yes-responses in each group of items.

For completeness, we repeat the wordings of the six dilemmas. The first three were standard trolley dilemmas (Bruers & Braeckman, 2013), whereas the last three were developed for Dahl and Oftedal’s (2019) study.

Dilemma 1: *The switch*. A trolley is moving toward five people on the main track. You are standing at a switch. If you turn the switch, the trolley will be diverted to a sidetrack, but there is one person on this sidetrack. Turning the switch will result in that person’s death, and the five people on the main track will be saved.
*Should you turn the switch?*
Dilemma 2: *The bridge*. A fat man is standing on a bridge above the track. You can save the five people on the track below by pushing the fat man from the bridge in front of the trolley, so that the trolley will be stopped by his heavy weight. The fat man will die, and the five people will be saved.
*Should you push the man?*
Dilemma 3: *The loop.* As in the first dilemma, you are standing at a switch. But this time the sidetrack turns back onto the main track. If there is no one on the sidetrack, the trolley will still move onto the main track and will kill the five people. But on the sidetrack is a fat man. So, if you turn the switch, the fat man will block the trolley, save the five people on the main track, but die himself.
*Should you turn the switch?*
Dilemma 4: A research group has developed a vaccine against the contagious disease Alobe, which occasionally has large outbreaks in the central African country Manigua. The effect of the vaccine (the immunity) is short lived, but mass-vaccination around an outbreak is expected to save thousands of lives in the future. In experiments, the vaccine triggers fatal heart failure in 3% of laboratory mice, but this side effect is unlikely for humans, who will receive a smaller dosage in relation to body weight. However, the Manigian authorities refuse to give approval of the vaccine, until its safety is established. The project will therefore test the safety of the vaccine on 1,000 consenting adults, but discontinue the testing in case of deaths. The participants will receive no compensation, and will most likely have no benefit from the vaccine, since the effect is short lived. One still expects subjects to volunteer, since it is inherent in the Manigian culture to take personal risk to save others.
*Is the study ethically justified?*
Dilemma 5: The same setting as above, except that the assumption about Manigian culture turns out to be incorrect. To recruit volunteers, the project will offer the Alobe vaccine bundled with a new vaccine against Malaria, which is otherwise not accessible. The Malaria vaccine is known to have a positive health effect that outweighs the risk of the Alobe vaccine.
*Is the study ethically justified?*
Dilemma 6: The same setting as above, but the research group argues that the subjects are autonomous individuals, and prefer to compensate them with two Manigian annual salaries, which is easier to administer than bundled vaccination. The subjects will be informed that this amount is sufficient to finance Malaria vaccination for their entire nuclear family at the local health station, if they choose to do so.
*Is the study ethically justified?*

The Trolley Score was defined as the number of yes-responses to Dilemmas 1 to 3, whereas the Vaccination Score was defined as the number of yes-responses to Dilemmas 4 to 6, giving both a range of 0 to 3. In addition to the six dilemmas, the collected data included gender only, to make the data set anonymous.

Our main hypothesis was the same as for the study on ethics committee members and staff (Dahl & Oftedal, 2019): that the Trolley Score would correlate with the Vaccination Score, with a two-sided test and significance level of 5%. We also wanted to compare additional dimensions of the responses, such as level of yes-responses between and in the different item groups, with those of the ethics committee members.

## Results

### Descriptive Statistics

There were 124 respondents, of which 113 delivered fully completed forms. Tables 1 to 3 give gender distribution, dilemma responses, and Trolley/Vaccination Scores, respectively.

**Table 1.** Gender Distribution.

Female	Male	Missing
89.1%	10.9%	3

**Table 2.** Distribution of Dilemma Responses.

Dilemma	No	Yes	Missing
Dilemma 1	5.7%	92.3%	2
Dilemma 2	51.2%	48.8%	3
Dilemma 3	13.3%	86.7%	4
Dilemma 4	75.6%	24.4%	5
Dilemma 5	77.1%	22.9%	6
Dilemma 6	85.7%	14.3%	5

**Table 3.** Distribution of Trolley and Vaccination Score.

Score type	0	1	2	3	Mean	Missing
Trolley score	5.9%	6.7%	39.5%	47.9%	2.29	5
Vaccination score	58.6%	25.9%	10.3%	5.2%	0.62	8

### Psychometric Properties

We used the Cronbach’s alpha statistic to measure reliability of the indexes (DeVellis, 2017). Cronbach’s alpha gives an estimate, on a scale from −1 to 1, of the internal correlation among items of an index. The alpha score of the Trolley index was .60, whereas the Vaccination index received a score of .55. Responsiveness was estimated using the entropy-based measure of information efficiency (Dahl & Østerås, 2010), which gives a measure of information content on a scale from 0 to 1. Information efficiencies of Dilemmas 1 to 6 were found to be 0.32, 1.00, 0.57, 0.80, 0.78, and 0.59, respectively. The Trolley index had an information efficiency of 0.78 and the Vaccination index 0.76, as given by the index response distribution (Table 2). The information contained by the two indexes are thus 78% and 76% of the theoretical maximum, which is acceptable. No external gold standards, by which to evaluate the validity of the indexes, are available in the current study, but the high response rate may show that most subjects understand the relevant problems as meaningful.

### Hypothesis Test

We found no statistically significant Pearson’s correlation (*p* = .302). The point estimate was 0.098, with confidence interval of [−0.089,0.285], which implies that the Trolley Score could only explain 1.0% of the variance in the Vaccination Score (which may as well turn out to be 0% as there was no statistical significance). We also merged the committee member data and the nurse data and evaluated the hypothesis on this larger data set of 181 cases. This gave an estimated correlation of .070, with a confidence interval of [−0.077,0.217] and a *p* of .35.

## Discussion

The psychometric properties were somewhat weaker for the nurse study compared with the ethics committee study, with Cronbach’s alpha levels below the recommended limit of .7. However, as discussed in Dahl and Oftedal (2019), this criterion is less applicable to indexes with as few as three items. It may also be related to a lower information efficiency of the nurse responses, which indicates a ceiling effect for the trolley scale and a floor effect for the vaccination scale. Still, the spread of the scores was large enough that a substantial correlation between them could be estimated statistically, had it been present.

The nurse study confirmed and strengthened the finding of a very weak, or nonexistent, correlation between trolley scores and vaccination scores, and the point estimate was even lower than for the ethics committees. Even merging the two data sets did not increase the support of the hypothesis, as the correlation estimate dropped further and the *p* value increased. It therefore seems that the lack of correlation between responses to trolley problems and responses to the vaccination scenarios is more general than indicated by Dahl and Oftedal (2019). Our results lend further support to studies that question the external validity of trolley problems (Bauman et al., 2014; Gold et al., 2014; Kahane, 2012, 2015; Wilson, 2016). It also strengthens the normative conclusion that one should be cautious in using trolley-based reasoning in discussions of vaccine trial ethics, in particular, and possibly medical research ethics in general.

The results of the specialist nurse study suggest, in accordance with Dahl and Oftedal (2019), two possible explanations. Either there is no utilitarian–deontological axis to be transferred from trolley dilemmas to related, more realistic dilemmas, or there is such an axis, but contextual factors influence the responses to the vaccine trial and dominate the result. In the trolley literature, one line of argument holds that trolley dilemmas do not reflect people’s utilitarian or deontological moral judgment. For instance, Kahane (2012) argues that people engage in a much richer and complex process when addressing moral dilemmas than only weighing utilitarian versus deontological solutions. A recent study by Bostyn et al. (2019) does suggest a correlation between how people respond to trolley dilemmas and how they respond to more realistic dilemmas. The realistic dilemmas in this study, however, were strictly monetary and had minimal context of the form: “should you take away a small random financial bonus from one person to distribute it equally to a group of people that originally had randomly received smaller bonuses?” The vaccine scenarios, however, have a richer context, and any link between the trolley problems and the vaccination scenarios appears to be dominated by other factors. Such factors could be that participants assign different weight to various aspects of the vaccine context, such as consent, moral status, and immediacy, rendering the data too noisy to give any measure of any transferable utilitarian or deontological orientation (Dahl and Oftedal, 2019). It is reasonable to assume this would be the case also for other scenarios involving risk, consent, incentives, uncertainties, and so on, suggesting that trolley dilemmas cannot be used as an indication of how people will judge complex research ethics scenarios.

An additional finding in Dahl and Oftedal (2019) was that the ethics committee members had a high willingness to push a man in front of a trolley to save five, compared with previous studies. In that article, it was speculated that this anomaly might be due to the ethics training received by committee members and staff. However, the present population of nurse students reported even higher willingness to push the man, without such specific training. One might speculate further whether the relatively high willingness to push the fat man is a tendency that can be found among health workers. Many of the ethics committee members are medical doctors, nurses, or have other health-related occupations and thus share the health worker status with the specialist nurses in the present follow-up study. This finding may well, however, have nothing to do with the membership of the suggested group and may be a general trait of, for example, Norwegians or Scandinavians.

The ethics committee members had a higher willingness to act in the trolley dilemmas than to allow the different versions of the vaccination projects, which was considered paradoxical, as the factors of risk–reward ratios and consent heavily favored vaccination (Dahl & Oftedal, 2019). The authors attempted to explain this phenomenon also by the subjects’ assumed predispositions regarding problematic research projects in general. However, the present study gave even more extreme results of the same kind, to the degree that the psychometric properties of the scales became less favorable due to floor and ceiling effects. In other words, the nurse specialist students were so willing to accept all trolley propositions/outcomes and unwilling to accept any vaccination propositions, that less information was present in their individual responses. This result indicates a more general tendency of being cautious with regard to the vaccine trials and not that this cautiousness is due to a particular predisposition of ethics committee members toward problematic research projects.

## Best Practices

The current follow-up study confirms that it is problematic to reason from ethics of trolley dilemmas to ethics of vaccination studies.

## Research Agenda

Considering the central role of trolley dilemmas in ethics and psychology, it is of interest to investigate whether people’s responses to trolley dilemmas can tell us something about their ethical judgment in different scenarios, in our case medical research ethics. On the background of the current study and Dahl and Oftedal (2019), it would be relevant to investigate responses in different groups and to extend the research by adding different medical research ethics problems. It would also be of interest to include dilemmas of varying contextual richness to test whether it is possible to find a “tipping point” where correlation can be found.

## Educational Implications

Trolley dilemmas are important in ethics education, and our study suggests that they should not be used as a basis from which to reason about vaccination ethics. When using trolley dilemmas as an educational tool, it is of relevance to teach and discuss their limited value with regard to providing information about how people would reason in more realistic situations.
